# Personality underground: evidence of behavioral types in the solitary subterranean rodent *Ctenomys talarum*

**DOI:** 10.7717/peerj.8490

**Published:** 2020-02-18

**Authors:** María Sol Fanjul, Roxana R. Zenuto

**Affiliations:** Grupo ‘Ecología Fisiológica y del Comportamiento’, Instituto de Investigaciones Marinas y Costeras, Universidad Nacional de Mar del Plata, Consejo Nacional de Investigaciones Científicas y Técnicas, Mar del Plata, Buenos Aires, Argentina

**Keywords:** Ctenomys, Subterranean rodents, Personality, Behavioral types, Solitary

## Abstract

**Background:**

Animal personalities have been studied in a wide variety of taxa, but among rodents, available studies are relatively scarce and have focused mainly on social species. In this study, we evaluated the existence of personality in the solitary subterranean rodent *Ctenomys talarum*. Specifically, we aimed to test individual differences in behavior that are stable over time and context in males of *C. talarum* captured in the wild.

**Methods:**

Our experimental design included two series of three behavioral tests each, carried out with a 35 day time interval. Each series included an Open Field test, a Social Encounter test, and an Open Field test with a predator stimulus.

**Results:**

Of the total recorded behaviors, 55.55% showed temporal consistency. Principal component analysis of consistent behaviors grouped them into four dimensions that explain inter individual behavioral variability, in order of importance: activity, socioaversion, boldness and exploration. Therefore, our results suggest that the concept of animal personality is applicable to *C. talarum* and the dimensions found are in accordance with the ecological and behavioral characteristics of this species.

## Introduction

Animal behavior is considered one of the most flexible traits (West-Eberhard, 2003). Contrary to the idea of unlimited behavioral flexibility, increasing evidence indicates that the individual expression of behavior is limited by underlying state-variables such as morphology, skill set to gather information, physiology or energy reserves (Sih et al., 2004; Sih, Bell & Johnson, 2004; Sih & Bell, 2008; Wolf & Weissing, 2012). This means that individuals can only modulate their behavioral responses in a limited range, or repertoire, rather than a full range of possible behaviors for the species (Sih et al., 2004; Mathot & Dingemanse, 2015). This phenomenon is known as personality, temperament or behavioral type (Réale et al., 2007). In terms of data, a given behavioral trait must show individual stability over time, and at different ecological contexts—for example, under predatory risk or reproduction—thus revealing differences between individuals in the population or species (Zipser, Kaiser & Sachser, 2013). However, temporal consistence of personality is expected to be higher in short than in long periods of time. During ontogeny, personality traits also change within individuals. These changes in temporal consistency may arise due to differential exposure to environmental conditions or to individual differences in developmental plasticity when individuals are exposed to the same conditions (Stamps & Krishnan, 2014; Liedtke et al., 2015). Personality variation may be maintained because there are multiple optima in different conditions, over time or space (Boon, Réale & Boutin, 2007), such as variations in predation pressure (Réale & Festa-Bianchet, 2003), food availability (Dingemanse et al., 2004), or social condition (Both et al., 2005).

To account for the different dimensions of personality, five traits have been proposed to be used as a tool in personality research. These traits are based on the ecological context in which they can be measured (Réale et al., 2007); first, the *bold* (or shy) reaction to risky situations, and second, the *exploratory* tendencies towards a new habitat or different objects. The general levels of *activity* are often difficult to separate from the boldness or exploratory motivation of an individual. The last two traits are related to the social environment and should vary according to whether the subject species is social or solitary: levels of *sociality*, as an association or aversion to conspecifics, and general *aggressiveness* to the presence of a conspecific (Réale et al., 2007). Additional research had incorporated the idea of behavioral syndromes, describing behavioral traits associated with each other (Sih et al., 2004; Sih, Bell & Johnson, 2004). Presumably, the origin of these associations could be related to common state variables, physiological pathways or requirements to display a behavior (Sih et al., 2004). In addition, this extends the concept to different areas, such as life history, developing the “pace of life syndromes” (POLs, Réale et al., 2010) or physiology, developing “coping styles” hypothesis (Koolhaas et al., 1999). Particularly, POLs predict a link among physiological traits along a slow–fast continuum of life history strategies. Within this framework, important studies have highlighted the key role of the link of locomotor performance and behaviors (i.e., exploration or predator avoidance) to understand the complex arrange of different phenotypes (Newar & Careau, 2018; Le Galliard et al., 2013).

Despite the evolutionary value of variability, behavioral ecology has long focused on an optimization approach that studies behavioral averages in a particular context (Mathot & Dingemanse, 2015) and considers suboptimal behaviors as “noise.” The present focus on the natural variability of populations, leads to a growing development of the theoretical framework on animal personalities and also to the increasing development of research on personality in a broad spectrum of animal taxa (see reviews Sih et al., 2004; Sih, Bell & Johnson, 2004; Van Oers et al., 2005; Bell, 2007; Réale et al., 2007; Biro & Stamps, 2010; Bergmüller, 2010). Rodents represent forty percent of mammalian species, and behavioral research in this group is abundant. It mostly uses laboratory breeding lines or long-bred captive colonies from a few wild captured parental lines, where genetic and phenotypic variation is substantially reduced (Blank, 1992; Lynch, Lynch & Kliman, 1989). Since animal personality focuses on the study of inter individual variations of natural populations (Zipser, Kaiser & Sachser, 2013), research on rodent personality is relatively scarce. Moreover, most of it comes from social species such as chipmunks (Newar & Careau, 2018; Martin & Réale, 2008), marmots (Petelle et al., 2013), squirrels (Dosmann, Brooks & Mateo, 2015) and bank voles (Šìchová et al., 2014). Research in solitary wild species was probably neglected due to logistic difficulties. Such difficulties are even more pronounced when it comes to studying behavior in subterranean species, for which no research on personalities has been conducted to date. Given this picture, the solitary subterranean rodent *Ctenomys talarum* (Talas tuco–tuco) provided an excellent opportunity to evaluate animal personalities. Although both sexes are territorial, only males utter a typical territorial vocalization (tuc–tuc) that informs potential intruders about the presence of the owner in a territory (Schleich & Busch, 2002). Evidence of scars along the necks in males (Zenuto, Lacey & Busch, 1999) suggests the occurrence of strong agonistic contests in nature. This aggressiveness is an important component in territorial and reproductive performance in males of *C. talarum*. During experimental contests, males modulate aggression according to rival identity, that is, neighbors or strangers (Zenuto, 2010) and aggressively fight each other establishing a hierarchy of dominance (Zenuto, Vasallo & Busch, 2002). As a result, dominant individuals monopolize reproductive activity and aggressively prevent other males from accessing mature females (Zenuto, Vasallo & Busch, 2002), resulting in a polygynous mating system (Zenuto, Lacey & Busch, 1999). When females were allowed to evaluate potential partners, they show preferences for dominant males (Fanjul & Zenuto, 2017) and those who are able to exclude intruders from their territories (Fanjul, Varas & Zenuto, 2018). Proactivity copying styles or bold personality is related to aggression, territorial control and active avoidance of negative stimulus (Koolhaas et al., 1999), which characterizes males in *C. talarum*. Hence, we aimed to test the existence of personality, that is, consistent individual differences in behavior that are stable over time and contexts, in wild-caught *C. talarum* males. Following Réale et al. (2007), we tested personalities according to four traits: *boldness* as risk-taking, *activity* as movement, *exploration* as the tendency to walk around and recognize the space, and socioaversion in relation to reactions shown by each male when confronted with a conspecific. We hypothesize that these four personality traits would be in accordance with the ecological and behavioral characteristics of male tuco–tucos. Temporal consistency was tested using a period of time representative for the life span of *C. talarum*. In addition, given that locomotor performance and behavior are closely related to survival in wild animals, maximal speed was tested for association with the potential dimensions of personality of *C. talarum*. We predicted that socioaversion and activity would be associated with locomotor performance due to its relevance for territorial and anti-predatory behavior. Finally, a common method for evaluating personality involves the use of a single test in a particular condition, assuming consistency in time and context, but its validity is questioned (Beckmann & Biro, 2013). Here we recorded behaviors during a short and simple test as a potential proxy of personality and evaluated its correlation with the different dimensions of personality assessed.

## Materials and Methods

### Animal capture and housing conditions

Capture, housing and handling conditions followed procedures previously described by Fanjul & Zenuto (2017) and by Fanjul, Varas & Zenuto (2018). Specifically, wild adult *C. talarum* males were live-trapped at coastal grasslands near Mar Azul, Buenos Aires province, Argentine (37°18′ 26″ S 57° 02′ 30″ W). A total of 21 males (Mean ± SD: 174.19 ± 24.36 g) were caught during the reproductive season (between June and December) in 2017. This species is a medium-sized rodent (adult males range between 120 and 220 g). Males do not undergo regression of their testes after attaining reproductive maturity and contain sperm in their epididymes year-round (Malizia & Busch, 1991). Tube-shaped traps were inserted into animal’s burrow systems showing fresh surface mounds indicative of recent digging activity. We transported all animals to the laboratory, where each tuco–tuco was individually housed in a plastic cage (42 × 34 × 26 cm) with a wire-mesh lid and wood shavings for bedding. We fed them daily with fresh food (carrots, sweet potatoes, catalogna chicory, corn, mixed grasses and sunflower seeds) ad libitum to secure water provision since *C. talarum* does not drink free water. All individuals were maintained in the same animal room where temperature and photoperiod were automatically controlled (25 ± 1 °C; breeding season 14 L:10 D). The experiments were carried out from 9 AM to 14 PM, since *C. talarum* individuals show an asynchronous and arrhythmic activity pattern (e.g., high interindividual variation in activity bouts were detected, as well as the absence of a clear concentration of activities in defined periods of time during a 24 h cycle), both in laboratory and field conditions (Luna, Antinuchi & Busch, 2000; Cutrera et al., 2006). At the end of the experiments, the animals were returned to their site of capture. We used disposable gloves in all instances of sample collection and during the experimental trials. All equipment used during the study was washed with tap water and odorless glassware cleaner, wiped with 95% ethanol and allowed to air dry to ensure that no trace odors from previous trials remained. The mean time of residence of the animals in the lab was 2 months, and were subjected to only one test a day for each behavioral series. As in previous studies dealing with antipredatory behavior in *C. talarum*, we used a domestic male cat as scent donor (Brachetta, Schleich & Zenuto, 2015, 2016).

### Behavioral tests

Our experimental design included two series of three behavioral tests each, carried out 35 days apart, which allows us to record two observations for each behavior. Given that individuals of *C. talarum* live approximately 2 years (Busch et al., 1989), the time period between both series represented 4.79% of the life span of the species and constituted a considerable time-lapse to evaluate the stability of a behavior (Roberts & DelVecchio, 2000; Zipser, Kaiser & Sachser, 2013). Although it would seem not like much time, the period used in relation to the life span of the tucos, is equivalent, in comparative terms, to 3 years for a man (considering a human life expectancy of 69 years). In order of execution, each series involved an Open Field test (OF-representing an unknown area to explore), a Social Encounter test (ENC-representing a neutral unknown area with the presence of a conspecific), and an Open Field test with a predator stimulus (OFp-representing a known area with a threatening stimuli). Tuco–tucos were allowed to habituate for 5 days to captivity before the first behavioral series began. Before the tests, subjects were individually placed in a Perspex box with bedding (soiled shavings) from their own home cage and covered with a black cloth to minimize disturbance. Subjects were allowed to habituate to this box for 1 h before the test began (see Zenuto & Fanjul, 2002). During each test, subjects were allowed to enter the test apparatus at will, with a maximum waiting time of 30 min (maximum latency). Values of zero were recorded in the cases where individuals did not show a particular behavior. We have no missing data. The second series started 35 days after the last test of the first series was completed. All trials were recorded using a digital HD Handycam (Sony HDR-XR100) and evaluated later. The recorded behaviors were detailed in Table 1. After each test, subjects were returned to their own home cage.

**Table 1:** Behaviors measured at each test, characterization and consistency. Temporal consistency (Spearman’s correlation *r* and *p* values), sample variation (coefficient of variation and range) and tests of habituation Univariated Mixed Models) of behaviors recorded in each test (Open Field, Social Encounter and Open Field with predator odor), with the corresponding personality trait, attributed according to the test context following Réale et al. (2007) criteria: Boldness (B), Exploration tendencies (E), Activity levels (A) and Sociality (S). Selected behavioral responses to include in PCA analysis are shown in bold typeface.

Test	Behaviors recorded	Personality trait	Consistency *r* (*p*)	Variability CV (range)	Habituation *F*-value (*p*)
Open Field (OF)	**Latency to enter the OF**	E/B	**0.506 (*p* = 0.019)**	1.158 (5–1800)	0.00011 (0.9917)
	**Number of times the animal entered the OF**	E	**0.522 (*p* = 0.015)**	0.646 (0–14)	1.62988 (0.2163)
	**Total number of squares traveled in OF**	A	**0.560 (*p* = 0.008)**	0.634 (0–857)	0.03914 (0.8452)
	**Total time spent in the OF**	A	**0.696 (*p* = 0.000)**	0.563 (0–596)	1.30993 (0.2659)
	**Time spent walking in the OF**	A/E	**0.657 (*p* = 0.001)**	0.621 (0–448.24)	0.04384 (0.8363)
	**Time spent in the center of OF**	B	**0.535 (*p* = 0.012)**	0.759 (0–104.14)	0.19337 (0.6648)
	**Total frequency of rearing behavior in the OF**	E	**0.471 (*p* = 0.030)**	0.654 (0–232)	0.57046 (0.4589)
	Total frequency of scratching OF	E/A	0.173 (*p* = 0.448)	0.958 (0–124)	0.68461 (0.4178)
Social Encounter test (ENC)	Latency to enter ENC		0.416 (*p* = 0.060)	1.160 (4.11–1800)	2.29278 (0.1456)
	**Total frequency in which the subject entered a neutral arena with a conspecific**	E/B	**0.668 (*p* = 0.000)**	1.160 (0–12)	0.87704 (0.3602)
	Time in neutral arena and near a conspecific	S	0.420 (*p* = 0.057)	0.589 (0–531)	0.03618 (0.8511)
	Time spent scratching mesh that separate a conspecific	S	0.200 (*p* = 0,379)	1.104(0–207.97)	0.71145 (0.4089)
	Total frequency of flee behavior	S	0.161 (*p* = 0.480)	1.583 (0–8)	4.285714 (0.0516)
	**Total frequency of sniffing a conspecific**	S	**0.533 (*p* = 0.012)**	0.700 (0–55)	5.90623 (0.0246)
	Total frequency of freezing behavior	S	−0.268 (*p* = 0.234)	1.372(0–6)	3.782723 (0.0660)
	**Total number of exposing their back to a conspecific**	S	**0.564 (*p* = 0.007)**	1.332 (0–21)	2.52900 (0.1275)
Open Field with predator odor (OFp)	Latency to enter OFp	E	0.767 (*p* = 0.000)	1.353 (5–1800)	2.18559 (0.1549)
	Total frequency the subject entered the OFp	B/E	0.395 (*p* = 0.075)	0.545(0–19)	5.92957 (0.0244)
	Total number of squares traveled in OFp	E/B	0.670 (*p* = 0.000)	0.595(0–1127)	0.27012 (0.609)
	**Total time spent in the OFp**	B/E	**0.442 (*p* = 0.044)**	0.387 (0–600)	1.30993 (0.2659)
	Time spent walking in OFp	B/E	0.275 (*p* = 0.223)	0.439 (0–507.55)	0.01774 (0.8954)
	Time spent in the open area (center) near predator odor	B	0.231 (*p* = 0.308)	0.570 (0–190.43)	1.40806 (0.2493)
	Total frequency of rearing behavior in OFp	B/E	**0.643 (*p* = 0.001)**	0.573 (0–217)	0.43963 (0.5149)
	Total frequency of scratching OFp	B/E	**0.692 (*p* = 0.000)**	0.826 (0–116)	4.74460 (0.0415)
	Time spent close or touching predator odor	B	0.208 (*p* = 0.361)	0.861 (0–154.48)	3.06117 (0.0955)
	Total frequency of sniffing predator odor	B	0.298 (*p* = 0.185)	0.526 (0–21)	1.62989 (0.2163)
	**Time spent sniffing predator odor**	B	**0.371 (*p* = 0.096)**	0.7321 (0–64.38)	5.43361 (0.0303)

*Open Field* (OF) consisted of a dark acrylic box (100 cm × 100 cm × 35 cm height) connected to a Perspex box covered by a black fabric to be perceived as a refuge. The floor was divided into 25 identical squares marked by lines for analysis purposes. The subjects entered the open field through a hole placed in the middle of one of the walls (see further details in Brachetta, Schleich & Zenuto (2015)). We defined “center” as the inner 3 × 3 squares of the field. Each test lasted 10 min once the subject first entered the OF device.

*Social Encounter test* (ENC) allowed us to evaluate the behavior of each subject exploring a neutral arena where a male conspecific was confined in a Perspex tube provided with a wire mesh at each end. This device allowed the test subjects to use chemical, vocal, and visual communication channels to evaluate a conspecific and modify their behavior, but avoiding the aggressive interactions characteristic of this solitary and highly territorial species (see Fanjul & Zenuto, 2013). Each test lasted 7 min after the subject’s first entry into the neutral arena.

*Open Field with predator odor* (OFp). Tuco–tucos were exposed to olfactory cues indicative of the presence of a predator using the OF in the first test, but with the addition of a predator odor source placed in the central square (Brachetta, Schleich & Zenuto, 2015). Previous studies showed that the exposure of tuco–tucos to odors from a cat provoked several changes in their behavior (see Brachetta, Schleich & Zenuto, 2015, 2016, 2019). A piece of cloth (6 cm × 6 cm) impregnated with cat fur odor (obtained after allowing a cat to use the cloth to rest on it for a period of 7 days before the experiment) was placed in a plastic Petri dish (diameter: 11 cm) covered with a wire mesh. Odor samples were stored at 4–6 °C in sealed plastic bags until use. Each test lasted 10 min.

### Behavioral data and statistical analyses

During the three tests performed, we recorded the behaviors detailed in Table 1. These were classified into four behavioral traits according to the context of the test-novelty, risk, and presence of a conspecific- and following Réale et al. (2007) criteria: boldness, exploration, activity and socioaversion.

Temporal consistency is one of the criteria that must be evaluated to consider that behavior meets the definition of personality. To test this, we used bivariate Spearman’s rank cross-correlations (Sigmaplot 14; Systat Software Inc., Chicago, IL, USA) between the first and second series of observations for each behavior (Zipser, Kaiser & Sachser, 2013; Horváth et al., 2016; Mota-Silva et al., 2010). As measures of relative variability that characterize the behaviors evaluated, the coefficients of variation (for each individual data from two test-series were averaged, then CV was calculated) and range for each of them are reported. The experimental design proposed in this study allowed not only the evaluation of the temporal consistency, but also the context consistency, since the Open Field test was used in two different contexts (with and without predator stimuli). Then, context consistency was tested using bivariate Spearman’s rank cross-correlations (Sigmaplot 14; Systat Software Inc., Chicago, IL, USA) over the behavioral records obtained for each individual (data from two test-series were averaged) in two different tests: OF without predator stimuli and with predator stimuli. Finally, to control for the possible effects of habituation, differences in each behavior were evaluated through a mixed-effect model, with the fixed factor Time and the random factor “individual.” This model was fitted using the “*nlme”* package (Linear and Nonlinear Mixed Effects Models, Pinheiro et al., 2019) of the “R” software (R Core Team, 2019). We checked by visual inspection that the statistical assumptions of homoscedasticity and normality were not violated. In cases in which the assumptions were not met, we transformed the data (either with square root or logarithm +1) and in some extreme cases where even with data transformation assumptions were not met we structured the variance with the VarIdent function (Zuur et al., 2009; see Table S1).

Twelve consistent variables (representing each one of the four personality traits in the most balanced way as possible) were included in the principal component analysis (PCA); this also reduced the variable number: sample size ratio and then contributed to the robustness of the analyses. Only one of these variables, *time spent sniffing predator odor*, failed in its temporal consistency but was incorporated due to the biological relevance of this information about the boldness trait (Table 2). For each behavioral variable, we averaged individual behavioral records of both observations (one for each series). We applied PCA in “R” software, a standard multivariate methodology previously used to study animal personalities (Martin & Réale, 2008; Šìchová et al., 2014; Freret-Meurer & Alves, 2018). PCA allowed us to reduce a large number of correlated variables into a smaller number of orthogonal variables, as scores of principal components, and retain most of the variance explained by original data. We tested sampling adequacy with “KMO” function from “*psych*” package (Revelle, 2018) of “R” program (R Core Team, 2019). PCA were performed using “*princomp”* function, a built-in function of “R” program, using correlation matrix (R Core Team, 2019). Principal components were retained to explain a minimum of 85% of the variance (Jolliffe, 2002) and according to their biological relevance (Sung et al., 2015; Worthington & Whittaker, 2006).

**Table 2:** Principal component’s loadings of each behavior. Variable loadings of retained principal components obtained from PCA of each individual behavioral data performed on a correlation matrix. Bold loadings indicate those variables that contributed the most to each component.

	Principal components			
Behaviors	1	2	3	4
Latency to enter the Open Field (OF)	**0.325**			**0.360**
Number of times the animal entered the OF	−0.266	0.281		**−0.556**
Total number of squares traveled in OF	**0.325**	0.265		0.195
Total time spent in the OF	**−0.352**			0.121
Time spent walking in the OF	**−0.335**	0.128	0.146	0.217
Time spent in the center of the OF	−0.285	−0.137	**0.447**	
Total frequency of rearing behavior in the OF	−0.309	**0.331**	−0.205	
Total frequency in which the subject entered a neutral arena with a conspecific	−0.293	−0.203		**−0.369**
Total number of exposing their back to a conspecific	−0.164	**−0.621**		
Total frequency of sniffing a conspecific	−0.273	**−0.443**	−0.269	
Time spent in the OFp	−0.270	0.139	**−0.301**	**0.537**
Time spent sniffing predator odor	−0.209	−0.235	**0.740**	0.180
***Importance of components:***				
***Standard deviation***	**2.758**	**1.232**	**0.940**	**0.863**
***Cumulative proportion of variance explained***	**0.634**	**0.760**	**0.834**	**0.896**

### Personality, locomotor performance and proxies

Principal component analysis scores of the retained components were used in further analyses to evaluate the association of each component with maximal locomotor speed. The maximal speed was measured as the spontaneous acceleration performed by a subject when running (without stop) in a straight line from one corner of the OF to the next corner. We recorded the speed reached by a subject in three events and then selected the fastest. Then, bivariate Spearman’s rank cross-correlations between the scores and maximal speed were performed.

The validity of a simple 3 min test as a proxy to assess personality in *C. talarum* males was also evaluated. Once captured in the field, the animals were transported in plastic tubes provided with grasses as food and paper as bedding. Immediately after we had arrived at the lab facilities, each tuco–tuco was placed in its individual home cage containing clean wood shavings and a refuge consisting of half of a terracotta pot. The behavior of tuco–tucos during the first 3 min in their own cage was recorded using a digital HD Handycam (Sony HDR-XR100) and evaluated later. Several behaviors were recordered in the subsequent analysis of these recordings: *time walking*, *time spent in the refuge*, *time displaying freezing behavior*, *time resting*, and *frequency of sniffing* the new habitat. Then, we performed bivariate Spearman’s rank cross-correlations (Zar, 2010) of these behaviors vs. PC scores.

#### Ethical note

We adhered to the 2012 Revised International Guiding Principles for Biomedical Research Involving Animals developed by the Council for International Organizations of Medical Sciences (CIOMS) and the International Council for Laboratory Animal Science (ICLAS). All procedures were revised and approved by the local committee for animal use and care in research (Comite Institucional para el Cuidado y Uso de Animales de Laboratorio, FCEYN UNMDP RD 467-17). Field sampling was approved by Dirección Provincial de Fiscalización y Uso Agropecuario de los Recursos Naturales, Ministerio de Asuntos Agrarios de la Provincia de Buenos Aires (File Number 22500).

## Results

A total of 27 behaviors were recorded in the three tests performed in two series. Most of them showed high interindividual variance. From the two observations of each behavior obtained 35 days apart, we identified 15 behaviors (representing 55.55% of the total behaviors recorded) that showed temporal consistency in the response throughout time (Table 1). Results of context consistency showed significant correlations for *total distance traveled* (*r* = 0.905, *n* = 21, *p* < 0.000002), *total time in OF* (*r* = 0.605, *n* = 21, *p* = 0.0006), and *frequency of rearing behavior* (*r* = 0.825, *n* = 21, *p* = 0.0000002).

The index of sampling adequacy was calculated as a KMO factor presenting an overall MSA = 0.74. In PCA we retained four major axes explaining the 89.6% of the total variance (see Table 2). The first principal component (PC1) included a group of variables related to activity, where *latency to enter the* OF *time spent in OF*, *time spent moving in OF*, and *distance traveled in OF* were those that loaded highest. The second principal component (PC2) loaded significantly with variables related to socioaversion, such as *exposing back to a conspecific*, *interacting with a conspecific*, and *rearing behavior in OF*. The third principal component (PC3) included variables measuring boldness, where *time spent in the center of OF* and *time spent sniffing predator’s odor*, were those that loaded highest. The fourth principal component (PC4) included variables related to exploration, where *time spent in OFp, latency to enter the OF, number of visits of the OF*, and *number of visits in ENC* were identified as those that loaded highest.

**Figure 1:**
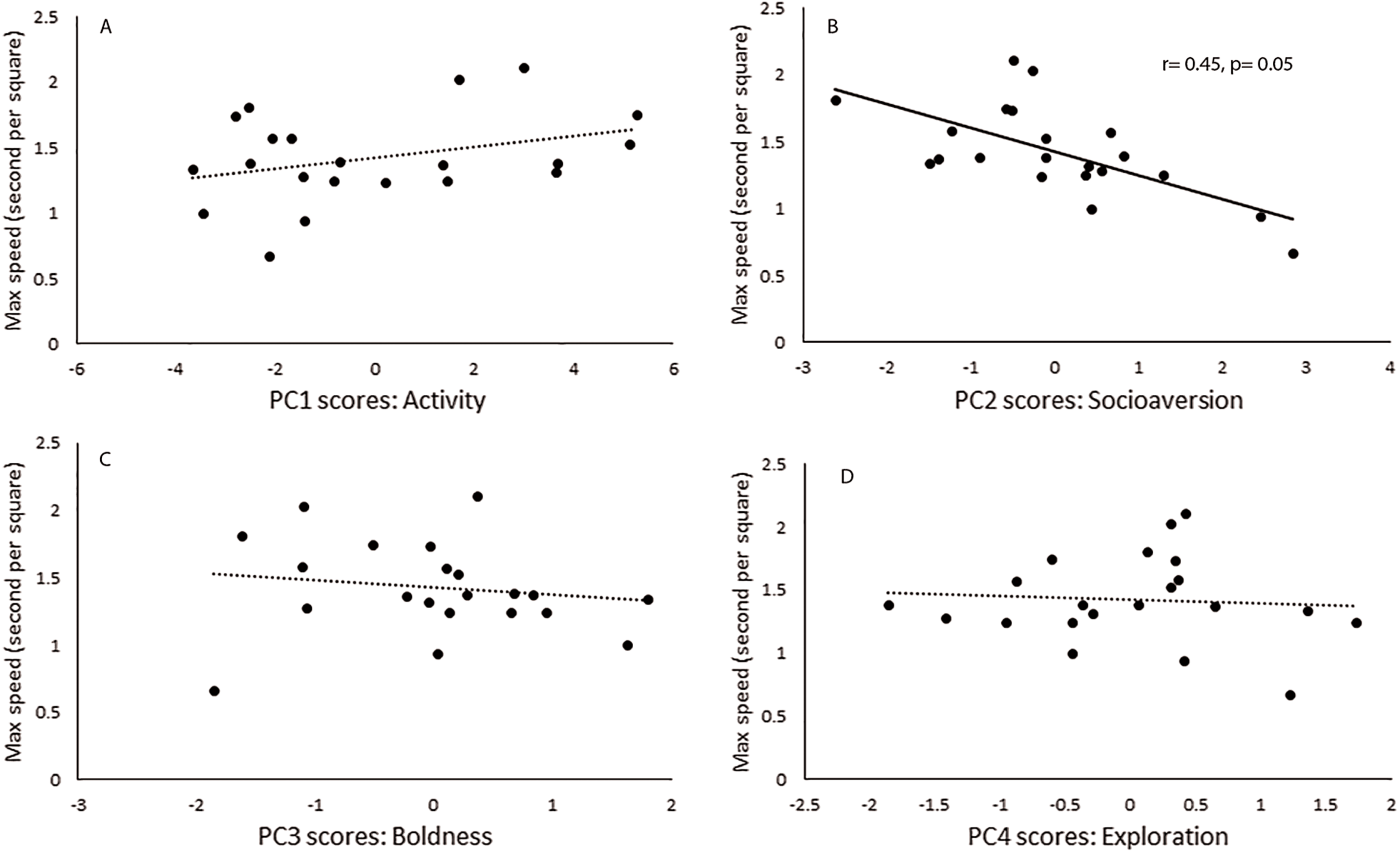
Behavioral traits and locomotor performance. Relationship between principal component scores (A) Activity, (B) Socioaversion, (C) Boldness and (D) Exploration scores, and maximal running speed for each male of *Ctenomys talarum* (*n* = 21). Full line and *r* correlation coefficient for Spearman Rank correlations along to *p* values are reported for significant relationships.

Maximal speed was found to be negatively correlated only to the scores of PC2, which loaded mainly with socioaversion behavior (Fig. 1). The first response of individuals to their new home cage was evaluated in a short and simple test. Some of the recorded behaviors were found to be related to personality components. *Resting behavior* was identified associated with the activity component, which characterized PC1, whereas *sniffing the substrate* was associated with socioaversion, which corresponded to PC2 (Table 3).

**Table 3:** Relationships between principal components scores and potential proxies of personality. Relationships of behaviors recorded in a single test to evaluate for personality proxies and principal components scores. In the table we reported *r* correlation coefficient for Spearman Rank correlations along to *p* values, between parentheses.

Behaviors	Principal components scores			
Recorded	**PC1-activity**	**PC2-socioaversion**	**PC3-boldness**	**PC4-exploratory**
Movement	−0.024 (0.982)	−0.353 (0.114)	−0.192 (0.398)	0.370 (0.096)
In the refuge	−0.034 (0.877)	−0.279 (0.216)	0.071 (0.754)	0.086 (0.703)
Freezing behavior	−0.124 (0.585)	−0.279 (0.216)	0.071 (0.754)	0.086 (0.703)
Resting	0.513 (0.017)	0.205 (0.367)	0.104 (0.649)	0.175 (0.445)
Sniffing	0.196 (0.389)	−0.458 (0.036)	−0.075 (0.754)	0.66 (0.771)

## Discussion

With this report, a solitary subterranean rodent is added to the growing number of species showing animal personalities, and is also the first evidence of personality characterization in the genus *Ctenomys*. The maintenance of consistent inter individual differences in behavior, or personality, in populations is proposed to persist because multiple optima exist within a single environment over time and space (Sih, Bell & Johnson, 2004). In this sense, the underground environment provides conditions of physical stability to its occupants, as well as protection against predators. However, differences in biotic and abiotic parameters between geographic regions and habitats make underground mammal species an important example of evolutionary convergence but also of adaptive divergence (Nevo, 1999; Reig et al., 1990). Studies carried out in *C. talarum* along its distribution range showed that populations diverge as consequence of the interplay of changes in the habitat, genetic structure and demography at different temporal and spatial scale (Cutrera, Lacey & Busch, 2006; Mora et al., 2013). Such dynamic processes of change operating at the individual level may favor the origin and maintenance of variations in behavior, that is, personalities (Sih et al., 2004; Sih, Bell & Johnson, 2004).

### Behavioral consistency

More than 50% of the behaviors recorded were consistent in time. From those, consistency shown was high, varying from 0.44 to 0.69 (Spearman correlations *r*), considering that the values reported in the literature are frequently lower (see review by Bell, Hankison & Laskowski (2009)). In addition, exploratory behaviors were identified in all contexts and with higher consistency, ranging from 0.6 to 0.9. Studying the consistency of traits over a long timescale makes more sense from a life history perspective. However, these studies are scarce (Réale et al., 2010), probably due to logistic limitations, or because it is difficult to find consistency over an extended period of time in which animals suffer changes in relation to different biological processes, such as maturation (Bell, Hankison & Laskowski, 2009) and environmental effects (Liedtke et al., 2015). In this regard, personality traits are proposed to develop according to requirements and constraints that are age-dependent (Petelle et al., 2013). In the present study, we evaluated consistency over a period of time that represents more than 4.5% of *C. talarum* life span, and behavioral consistency was detected in most of the behaviors associated to the four behavioral dimensions of personality considered (activity, socioaversion, boldness, and exploration; Réale et al., 2007).

Habituation is a decreased response of an individual to a stimulus to which it has previously been exposed. In this study, we found a habituation effect in few behavioral measures in repeated tests. The only two variables included in PCA that showed habituation were *total frequency of sniffing a conspecific* and *time spent sniffing predator odor*. For an individual to become habituated requires the development of memory about the stimuli presented repeatedly. The memory of odors from conspecifics lasted 7 days and it was extended for 14 days when the subject also interacted with the odor donor in Talas tuco–tucos (Zenuto, 2010). Given that information, and considering that our study implies a 35 day trial-tests period, the habituation effect found was not expected. Moreover, the habituation effect on antipredatory behavior after 5 days of exposure to predator odor was detected in *C. talarum* (Brachetta, Schleich & Zenuto, 2016) while no longer periods were assessed.

### Personality dimensions

From PCA of consistent behaviors, the first four components explained 85% of total variability comprising four behavioral traits: activity, socioaversion, boldness and exploration. From these components, the first two explained most of the variance of behavioral data. *Total time spent in the OF*, *time spent walking*, *the distance traveled*, and in the opposite way, the *latency to enter the OF* were grouped in PC1. Such behaviors are mainly related to the activity levels of the subjects, and to a lesser extent, to exploration. In nature, the activity of this species involves the patrolling of its individual burrows, repairing the tunnel systems, and the excavation of soil towards patches with suitable vegetation to feed. For males, patrolling its burrows would also have a territorial function that would contribute to deterring potential intruders, an important task for this species (Busch et al., 2000; Zenuto, Vasallo & Busch, 2002; Cutrera et al., 2006).

The *exposure of the back* and *sniffing conspecifics* were the main behaviors grouped by PC2, both clearly related to social evaluation and male–male interaction. By exposing their backs, tuco–tucos show their interest in avoiding a potential interaction or to initiate a contest with another male (Zenuto, 2010; Zenuto, Vasallo & Busch, 2002; Fanjul & Zenuto, 2017). In the opposing direction, *rearing behavior* is also grouped within PC2. Although it is often considered an activity/exploratory trait, it would also reveal an anxiety condition (Réale et al., 2007) associated with perceived risk in an open and novel environment for a subterranean species like *C. talarum*. As stated earlier, tuco–tucos are highly territorial, and the presence of another male implies a certain level of alarm due to the imminent risk of suffering injuries during an aggressive encounter and even more, the loss of exclusivity of its burrow system (Zenuto, Vasallo & Busch, 2002; Zenuto, 2010; Fanjul, Varas & Zenuto, 2018), which could generate the display of anxiety behaviors. The third component grouped behaviors related to boldness such as *time spent sniffing predator odor* and *time spent in the central area of the OF*, and in the opposing direction it also included the *time spent in OFp*. The identification of predators by potential prey is very important for survival. Once the presence of a predator is detected, antipredatory behaviors are triggered, in the form of direct avoidance of odor source, changes in space use, enhanced vigilance, and decreased activity (Dielenberg & McGregor, 2001; Apfelbach et al., 2005; Ylönen et al., 2006). Due to its underground way of life, the time of brief lapses that tuco–tucos spent on the surface searching for food or during dispersal could be perceived as particularly risky due to the presence of aerial and terrestrial predators (Busch et al., 2000). In accordance with this predation risk, previous studies showed that the exposure of individuals to odors from a predator (cat urine, feces and fur) provoked anxiety responses, decreased locomotor activity, avoidance behavior and changes in feeding behavior (Brachetta, Schleich & Zenuto, 2015, 2016, 2019).

Finally, PC4 grouped behaviors related to the tendency for exploration and curiosity such as the *number of visits to the OF* and *to the neutral arena with a conspecific*, the *total time spent in the OFp* and *the latency to enter the OF*. A characteristic of subterranean rodents like tuco–tucos is that access to distant food sources is achieved only by the extension of the galleries (Lacey, Patton & Cameron, 2000). In the case of *C. talarum*, it is known that it uses chemical cues of the vegetation to direct underground excavation and thus access to patches of vegetation that have been later used for feeding (Schleich & Zenuto, 2007). Thus, exploratory behavior could be related to the continuous extension of their burrow systems for foraging purposes. However, this last component explains a much smaller portion of the data variability than activity and socioaversion, which appear to be the main personality traits for *C. talarum*.

As stated earlier, animals are exposed to a wide variety of challenges in nature that compromise their survival. Behavioral responses follow these changes, which occur throughout individuals’ life and environment. Despite behavioral flexibility, individuals often show consistent differences in behavioral traits that can be explained by individual niche specialization (Araújo, Bolnick & Layman, 2011; Montiglio, Ferrari & Réale, 2013). As proposed for social and foraging behavior (Toscano et al., 2016; Montiglio, Ferrari & Réale, 2013), individuals could function as specialists, showing a portion of the entire behavioral repertoire of the population—or species—according to their use of specific ecological conditions. Although it is no clear whether personality and individual specialization would covary, or are causally related, this perspective provides a possible explanation for the maintenance of individual differences in behavior even though natural selection is expected to diminish these differences (Araújo, Bolnick & Layman, 2011; Montiglio, Ferrari & Réale, 2013). Given the life history traits of *C. talarum*, both temporal and spatial effects on personalities may have an important role in this species. Changes in behavioral traits associated with exploration and boldness during ontogeny are expected, differentiating juveniles and adults. In adult males, dominance and subordination relationships were identified (Zenuto, Vasallo & Busch, 2002). Dominant males monopolize reproductive activity, aggressively impeding other males access to mature females (Zenuto, Vasallo & Busch, 2002), and preference of females for dominant individuals was also identified (Fanjul & Zenuto, 2017). The roles of dominant and subordinate could also be related to the way in which males cope with predatory risk and obtain food resources, displaying different combinations of activity, exploration, socioaversion, and boldness. Once identified the dimensions of behavioral traits that explain interindividual behavioral variability in *C. talarum* males, may prompt new studies considering life history traits that will shed light on individual specialization in subterranean rodents.

### Personality, locomotor performance and proxies

Given *C. talarum* life history characteristics it is not surprising to find an association between running performance and socioaversion. For C. *talarum* males, intrasexual competence is high; individuals aggressively defend their territories and, as polygynous rodents, this ability is tightly related to the access to multiple females (Zenuto, Lacey & Busch, 1999; Zenuto, Vasallo & Busch, 2001; Fanjul & Zenuto, 2017). Engaging in a fight is costly, both in terms of energy and risk of injury. Therefore, decision making during a contest (whether to fight or not) depends on a complex array of factors, but mainly on the assessment of the quality of contestants and the ability to escape (“fight or flight” response) for which locomotor performance is crucial. The consequences of agonistic interactions for animals have been studied for a long time (Maynard-Smith & Price, 1973; Parker, 1974). Fitness of the contestants could be strongly affected by these interactions (Hardy & Briffa, 2013), via aggression-related physiological changes (Nelson, 2005) or by impaired access to mates, habitats or food resources (Clutton-Brock & Albon, 1979).

We have found two behavioral proxies from behaviors recorded during a quick assay at animals’ arrival at lab facilities. These measures were correlated to two behavioral dimensions, activity and socioaversion. Although the use of proxy measures is a common tool in behavioral research, but it is necessary to evaluate the validity of using a single test as a predictor of consistent traits to properly employ it (Beckmann & Biro, 2013). The relationship found is also in agreement with the dimensions of personality we have previously described and to the behavioral ecology of *C. talarum*. We have found that resting behavior is a good proxy of the activity dimension of personality. Additionally, the time devoted to *sniffing the substrate* is a good proxy of socioaversion. This last association underlies the central role of chemical communication in the social features of Talas tuco–tuco (Zenuto, Vasallo & Busch, 2001; Zenuto, 2010; Fanjul & Zenuto, 2013; Fanjul, Varas & Zenuto, 2018).

## Conclusions

To sum up, three main conclusions emerge from our work. First, we provide evidence of personality in the subterranean rodent *C. talarum*. We found great variability in the expression of behavioral traits in the population studied and, at the individual level, consistency of their display over time and in different contexts. The personality dimensions are related to the levels of activity and to the socioaversion of individuals, and to a lesser extent to the boldness and exploratory tendencies. Second, we found that locomotor performance was associated with socioaversion dimension of personality, probably in relation to the ability to escape or chase competitors. Thus, high territoriality and the polygynous mating system could have a great impact on the dimensions of personality maintained by natural and sexual selection in tuco–tucos. Third and last, we were able to find two proxies, such as time resting and sniffing the substrate related to activity and socioaversion dimensions, respectively. The last association emphasizes once again the importance of chemical communication for social interactions in *C. talarum*, as expected for rodents, and especially in a subterranean species where other communication channels are precluded (Francescoli, 2000).

The next step corresponds to assessing personality in females. Females are characterized by lower aggression, so avoidance and not confrontation characterize the relationships with conspecifics (Zenuto, Vasallo & Busch, 2002). Additionally, the effect of the reproductive condition (pregnancy, lactation, or both during postpartum ostrus) is expected to have implications in the expression of behavioral traits such as exploration and activity (Zenuto, Antinuchi & Busch, 2002).

## Supplemental Information

10.7717/peerj.8490/supp-1 Supplemental Information 1 Raw data.

10.7717/peerj.8490/supp-2 Supplemental Information 2 Script from analyses performed with program R.

10.7717/peerj.8490/supp-3 Supplemental Information 3 Principal component analysis (PCA) biplot of behavioral data. Behaviors were abbreviated as follows: Latency to enter the open field (latOF), Number of times the animal entered the OF (visitOF), Total number of squares traveled in OF (distOF), Total time spent in the OF (timinOF), Time spent walking in the OF (movOF), Time spent in the center of the OF (centrOF), Total frequency of rearing behavior in the OF (rearOF), Total frequency in which the subject entered a neutral arena with a conspecific (visitENC), Total number of exposing their back to a conspecific (backENC), Total frequency of sniffing a conspecific (sniffENC), Time spent in the OFp (timinOFp), and Time spent sniffing predator odor (tsniffOFp).

10.7717/peerj.8490/supp-4 Supplemental Information 4 Detail of transformation functions used to meet the assumptions of Mixed Models for each variable.

## Additional Information and Declarations

### Field Study Permissions

The following information was supplied relating to field study approvals (i.e., approving body and any reference numbers): Field sampling was approved by Dirección Provincial de Fiscalización y Uso Agropecuario de los Recursos Naturales, Ministerio de Asuntos Agrarios de la Provincia de Buenos Aires (22500).

### Data Availability

The following information was supplied regarding data availability: The raw data and scripts are available as a Supplemental File.
