# Supplementary material for: Personality underground: evidence of behavioral types in the solitary subterranean rodent Ctenomys talarum
Source: PeerJ. 2020 Feb 18;8:e8490. doi: 10.7717/peerj.8490 (PMC7034374; doi:10.7717/peerj.8490)
Supplement: Supplemental Information 2 [file peerj-08-8490-s002.docx]

Fanjul and Zenuto

#Univeariate mixed models

search()

detach(Datos)

Datos <- read.table("clipboard", header=TRUE, sep="\t", na.strings="NA", dec=",", strip.white=TRUE)

library(nlme)

attach(Datos)

## choose models

M1<-lme((tsniffOFp)~ tiempo, data=Datos, random = ~1|tuco)

plot(M1)

M1<-lme(sqrt(tsniffOFp)~ tiempo, data=Datos, random = ~1|tuco)

plot(M1)

M1<-lme(log(tsniffOFp+1)~ tiempo, data=Datos, random = ~1|tuco)

plot(M1)

anova(M1)

## Check the assumptions

plot(M1)

plot(resid(M1))

qqnorm(residuals(M1))

qqline(residuals(M1))

#variance strructure

vf1 <- varIdent(form=~1|tiempo)

#without transform

fm11 <- lme((distOFp)~ tiempo, weights=vf1, data=Datos, random = ~1|tuco)

plot(fm11)

qqnorm(residuals(fm11))

qqline(residuals(fm11))

anova(fm11)

##Homocedasticidad de varianza

plot(M1, which= c(1), col = 1, add.smooth = FALSE, caption = "")

##Normality

hist(resid(M1), xlab= "Residuals", main="")

qqnorm(residuals(M1))

qqline(residuals(M1))

anova(M1)

# PCA CTENOMYS TALARUM PERSONALITY

setwd("C:/Users/Win10/Dropbox/personalidad/analisis y datos")

data<-read.csv("C:/Users/Win10/Dropbox/personalidad/analisis y datos/pers4.csv", header = TRUE)

attach(data)

#define variables

x<-cbind(latOF, centrOF, timinOFp, tsniffOFp, visitOF, timinOF, rearOF, movOF, distOF, visitENC, interENC, backENC )

#Descriptive statistics

summary(x)

cor(x)

write.excel <- cor(x)

#indices of sampling adecuacy

library(psych)

KMO(x)

#PCA

pca1<-princomp(x, scores = TRUE, cor = TRUE)

summary(pca1)

#Loadings of PC

loadings(pca1)

#PLOTS

#Scree plot of eigenvalues

plot(pca1)

screeplot(pca1, type = "line", main = "Scree plot")

#biplot

biplot(pca1)

#scores of components

pca1$scores[1:21,1:12]

citation()

citation(“psych”)
