# Supplementary figures and images for: Personality underground: evidence of behavioral types in the solitary subterranean rodent Ctenomys talarum
Source: PeerJ. 2020 Feb 18;8:e8490. doi: 10.7717/peerj.8490 (PMC7034374; doi:10.7717/peerj.8490)

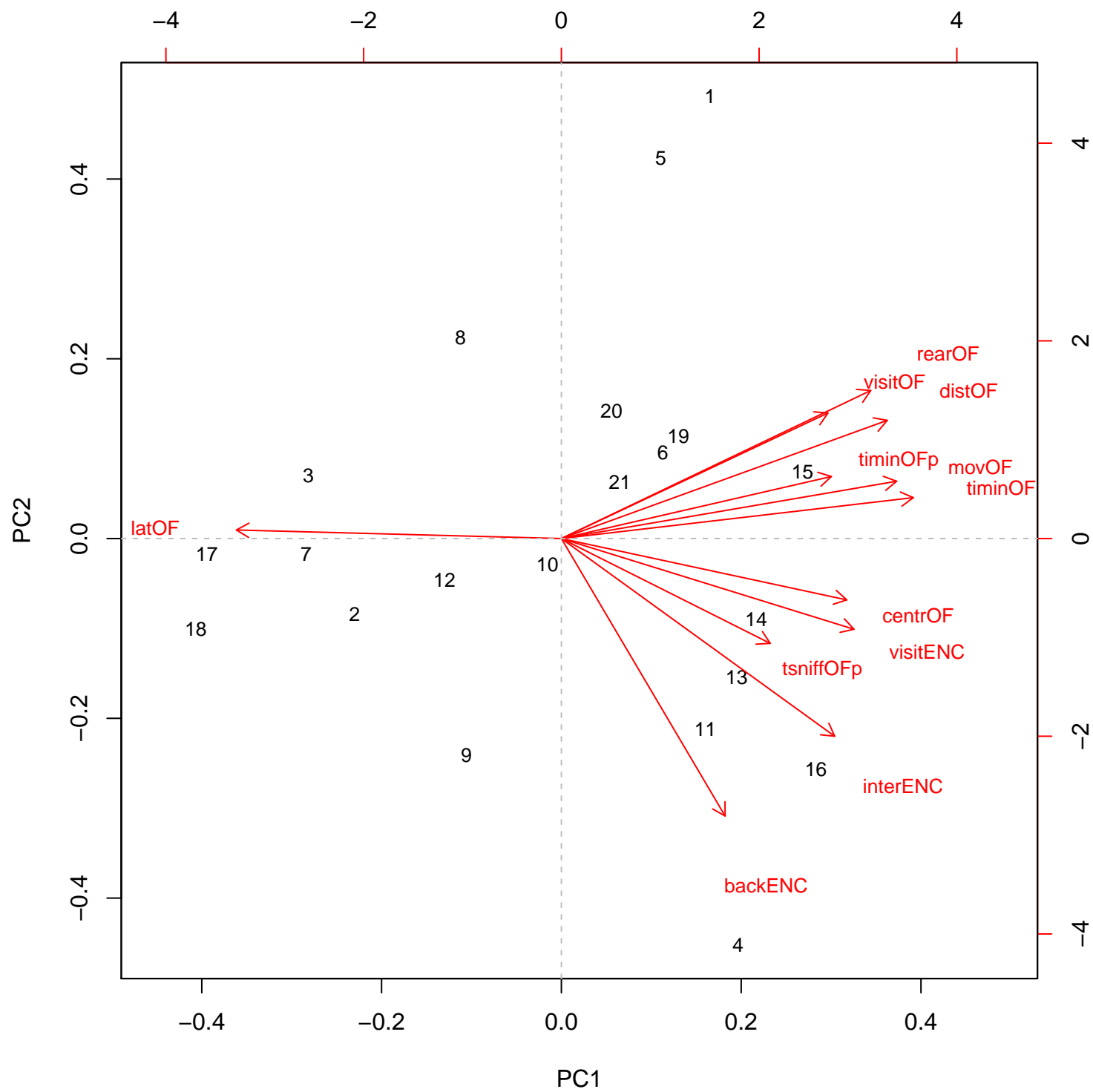

Supplement: Supplemental Information 3 — Behaviors were abbreviated as follows: Latency to enter the open field (latOF), Number of times the animal entered the OF (visitOF), Total number of squares traveled in OF (distOF), Total time spent in the OF (timinOF), Time spent walking in the OF (movOF), Time spent in the center of the OF (centrOF), Total frequency of rearing behavior in the OF (rearOF), Total frequency in which the subject entered a neutral arena with a conspecific (visitENC), Total number of exposing their back to a conspecific (backENC), Total frequency of sniffing a conspecific (sniffENC), Time spent in the OFp (timinOFp), and Time spent sniffing predator odor (tsniffOFp). [file peerj-08-8490-s003.pdf]
