# Supplementary material for: Personality underground: evidence of behavioral types in the solitary subterranean rodent Ctenomys talarum
Source: PeerJ. 2020 Feb 18;8:e8490. doi: 10.7717/peerj.8490 (PMC7034374; doi:10.7717/peerj.8490)
Supplement: Supplemental Information 4 [file peerj-08-8490-s004.docx]

Supplemental Table 1- Detail of transformation functions used to meet the assumptions of Mixed models for each variable.

|  | Transformation used | | |
| --- | --- | --- | --- |
| Variables | Square root | Log+1 | Structured variance |
| Latency to enter the OF | * |  |  |
| Number of times the animal entered the OF |  |  |  |
| Total number of squares traveled in OF |  |  |  |
| Total time spent in the OF |  |  |  |
| Time spent walking in the OF |  |  | * |
| Time spent in the center of OF |  |  | * |
| Total frequency of rearing behavior in the OF | * |  | * |
| Total frequency of scratching OF |  | * |  |
| Latency to enter ENC |  | * |  |
| Total frequency in which the subject entered a neutral arena with a conspecific | * |  |  |
| Time in neutral arena and near a conspecific | * |  |  |
| Time spent scratching mesh that separate a conspecific |  |  | * |
| Total frequency of flee behavior |  |  | * |
| Total frequency of sniffing a conspecific |  |  | * |
| Total frequency of freezing behavior |  |  |  |
| Total number of exposing their back to a conspecific | * |  |  |
| Latency to enter OFp |  | * |  |
| Total frequency the subject entered the OFp |  | * |  |
| Total number of squares traveled in OFp |  |  | * |
| Total time spent in the OFp |  |  |  |
| Time spent walking in OFp |  |  | * |
| Time spent in the open area (center) near predator odor |  | * |  |
| Total frequency of rearing behavior in OFp |  |  |  |
| Total frequency of scratching OFp | * |  |  |
| Time spent close or touching predator odor |  |  |  |
| Total frequency of sniffing predator odor | * |  |  |
| Time spent sniffing predator odor | * |  |  |
